# Extensive facial scarring after ablative laser resurfacing in a patient with frontal fibrosing alopecia

**DOI:** 10.1016/j.jdcr.2022.03.024

**Published:** 2022-03-26

**Authors:** Cong Sun, Davin Lim, Philip Bekhor

**Affiliations:** a Princess Alexandra Hospital, Brisbane, Queensland, Australia; b Cutis Clinic, Brisbane, Queensland, Australia

**Keywords:** alopecia, cosmetics, laser, FFA, frontal fibrosing alopecia

## Introduction

Frontal fibrosing alopecia (FFA) is a primary cicatricial alopecia with an unknown incidence in the general population. A single-center study in New York estimated the rate of FFA to be 5.41 in 100,000.^1^ It is a clinical subvariant of lichen planopilaris, which is the cause of 1.25% of all alopecia and up to 25% of cases of scarring alopecia.^2^ The fractional erbium laser is among the most frequently used resources in dermatology for facial rejuvenation.^3^ Laser resurfacing is regarded as a useful modality of treatment for acen scarring with a low risk for serious complications.^3^^,^^4^ We hereby present a case of extensive scarring after ablative laser resurfacing in a patient with undiagnosed FFA. This article aims to illustrate the likely mechanism of extensive scarring after ablative laser resurfacing in patients with FFA and thereby convey the importance of recognizing this condition in the daily practice of laser dermatology.

## Clinical record

**Fig 1:**
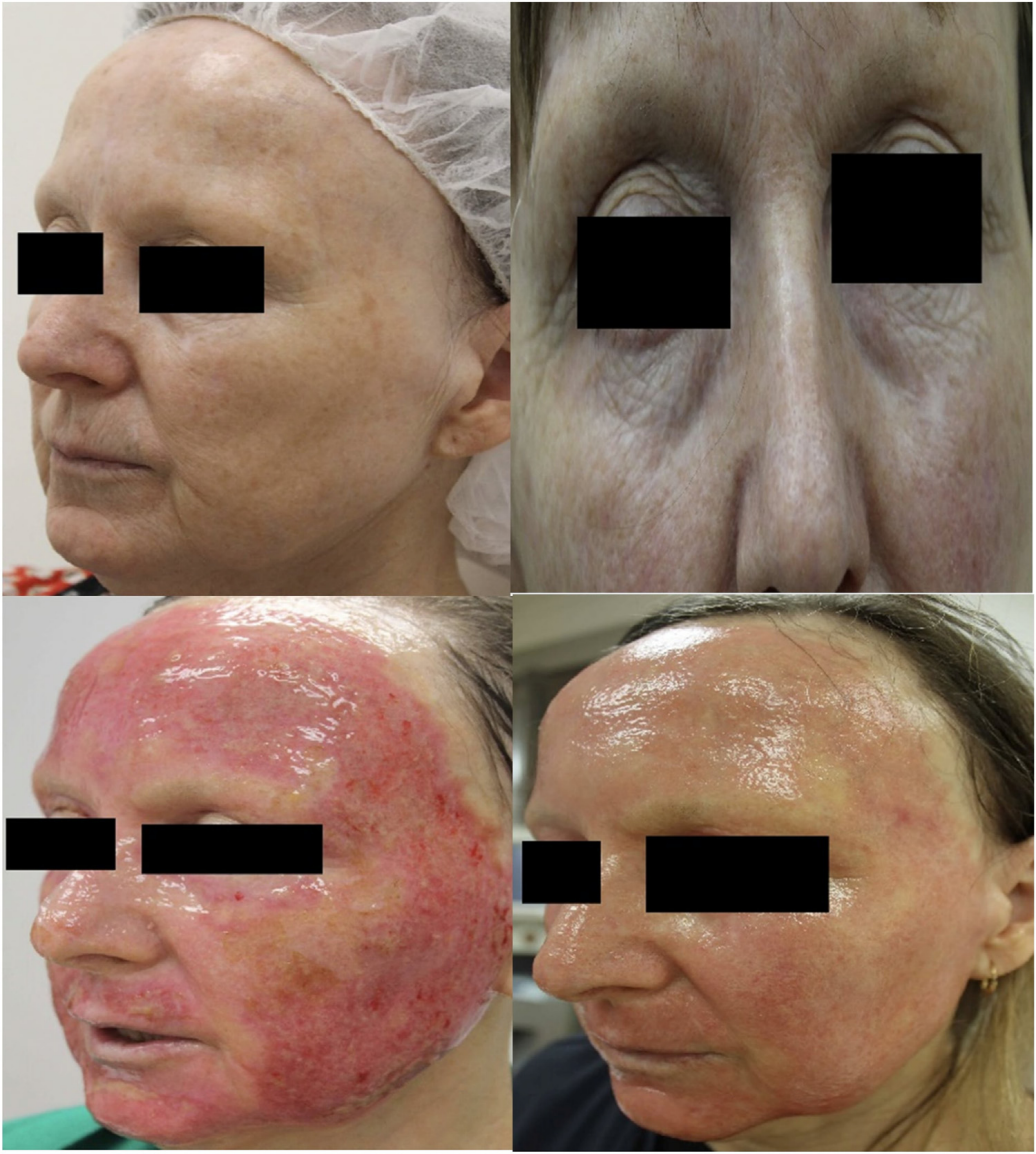
*Top left and right:* appreciable hyperpigmentation and rhytides; *Bottom Left:* day 2 post resurfacing procedure; *Bottom Right*: day 7 post treatment with complete re-

A 63-year-old woman presented with pigmentation and rhytides (Fig 1). The patient was otherwise medically unremarkable, with no previous history of viral or bacterial cutaneous infections or isotretinoin use, which are potential predisposing factors for postprocedural scarring and infection. Conservative fully ablative laser resurfacing was conducted for pigmentation and rhytides uneventfully, with an erbium 2940-nm laser with the following settings: 100-micron pass followed by 50-micron pass to the cheeks, single 80-micron pass to the eyelids, and 100-micron pass followed by 60-micron pass to the perioral area.

The aftercare routine consisted of 7 days of topical white soft paraffin and Cetaphil cleanser along with dressing and wound checks every 48 hours. On day 6, areas of erosion developed on the superior forehead, bilateral infraorbital areas, and superior lip and were extensively swabbed for bacteria and viruses, the results of which were all negative. Complete re-epithelialization was achieved by day 12, with residual erythema settling in the next 5 weeks (Fig 1).

Extensive global scarring over the treatment fields was noted at 7 weeks, with the most extensive involvement in the forehead and perioral area. Scar revision protocols were commenced, involving 3 weekly nonablative low-density CO_2_ laser (60-mj 3% density; 1 pass) treatments followed by topical triamcinolone suspension (40 mg/mL) under 3-hour occlusion. Methylprednisolone aceponate ointment was prescribed for pulse regimen twice daily for 3 consecutive days with 4 rest days. Silicone gel was applied to the perioral area for the thickest scars 3 times a day.

**Fig 2:**
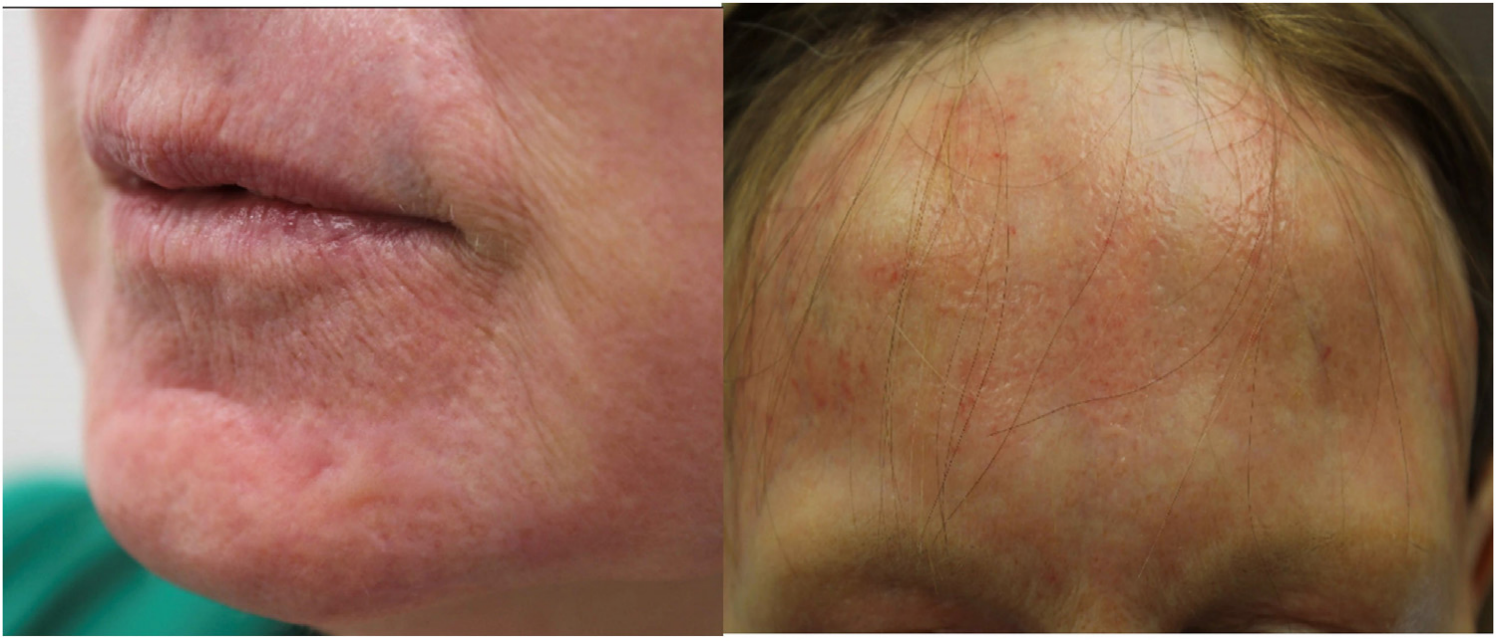
Extensive hypertrophic scarring of the forehead and perioral area at 7 weeks check-up post initial resurfacing procedure in the treatment field.

**Fig 3:**
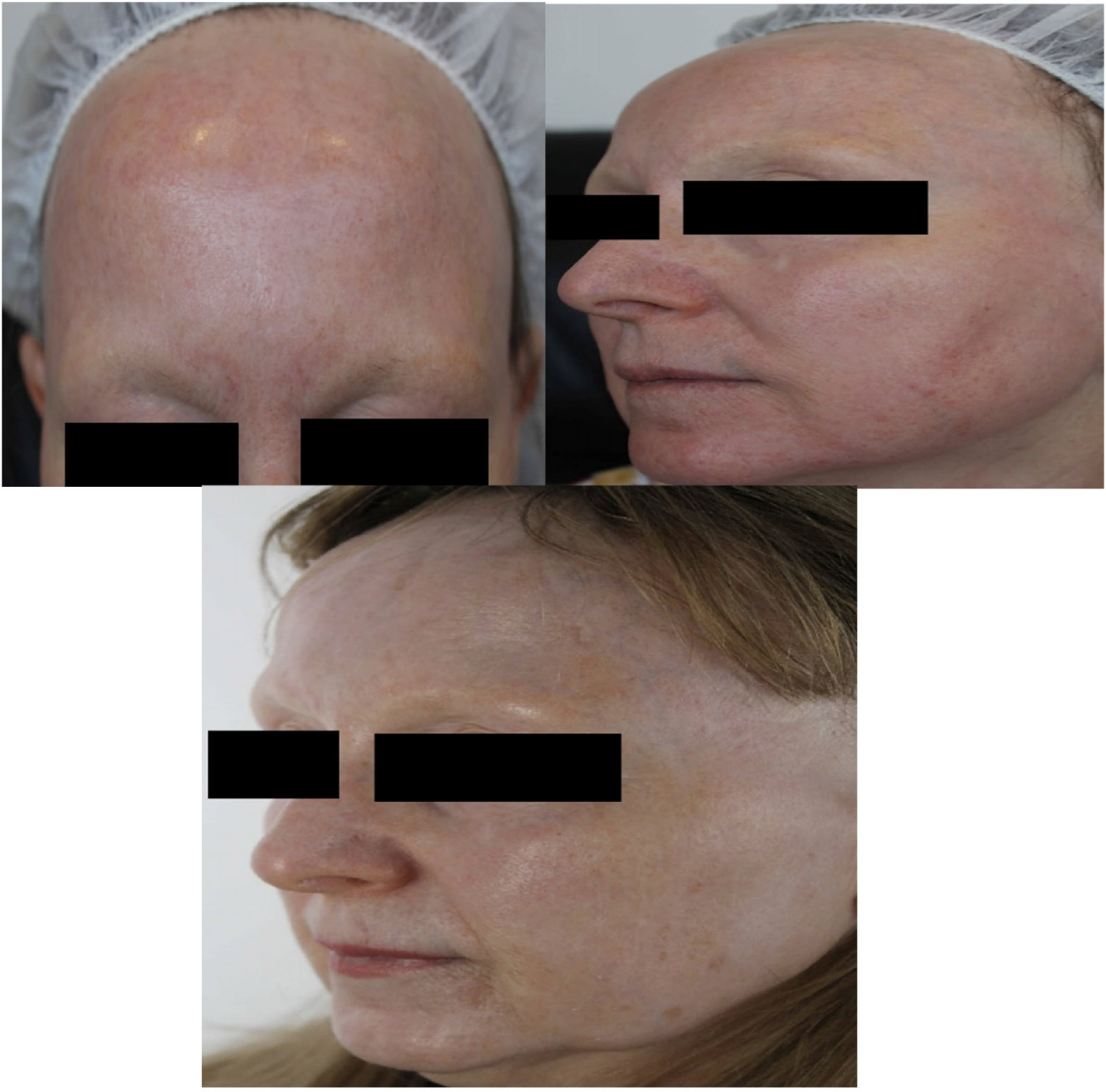
*Topleft andright:* complete resolution of hypertrophic scarring with the regimen of low density carbon dioxide laser, topical triamcinolone under occlusion, and topical silicone gel. *Bottom:* bilateral loss of eyebrow hair and loss of facial vellus hair.

**Fig 4:**
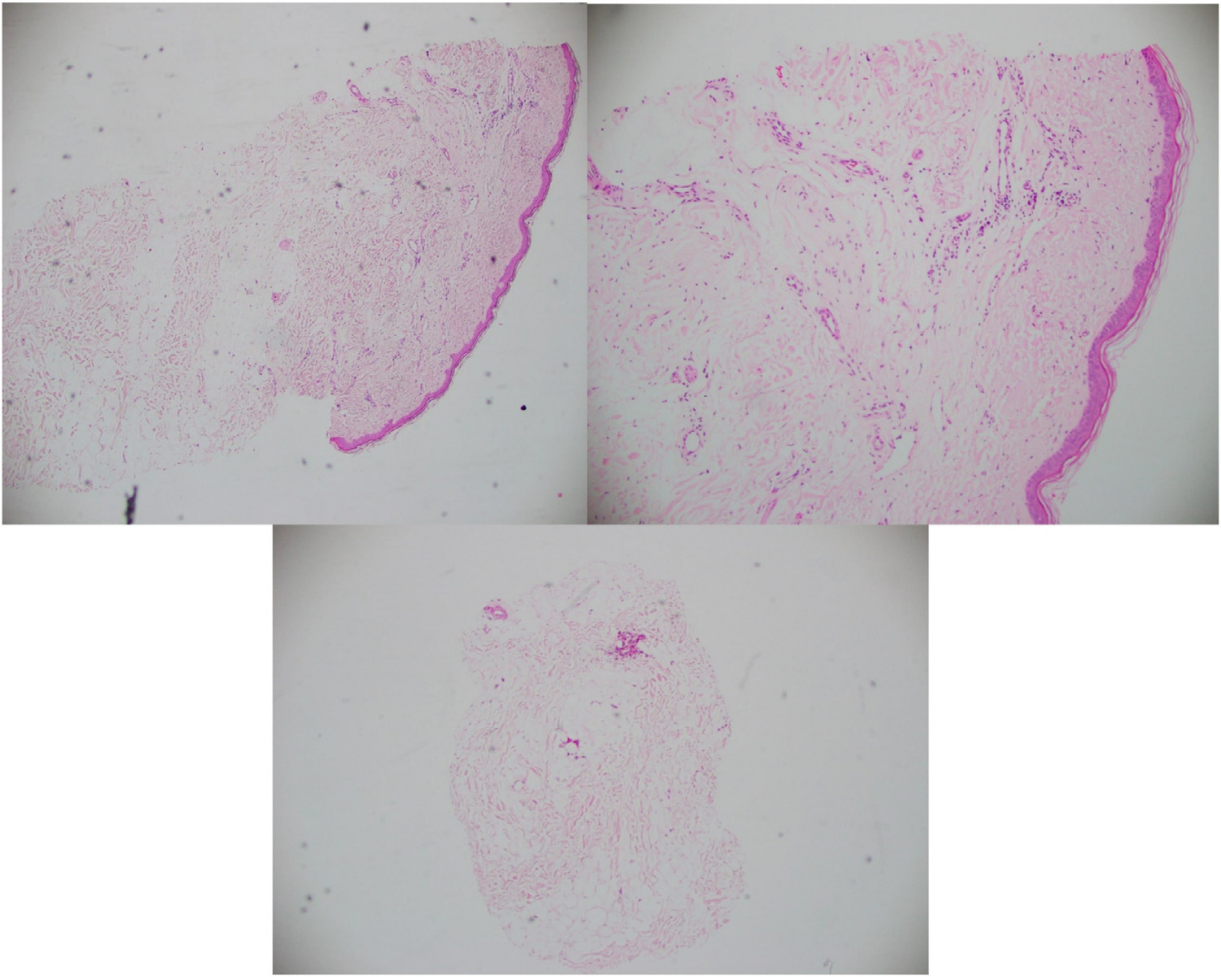
*Top left and right*: horizontal sections of 4mm punch biopsy from left preauricular superior. *Bottom*: vertical sections of 4 mm punch biopsy from right preauricular inferior. Both specimens show complete absence of hair follicle, eccrine and sebaceous glands with mild perivascular lymphocytic infiltrate consistent with end-stage scarring alopecia.

Sixteen weeks after the initial resurfacing procedure, the complete resolution of scarring was achieved (Fig 2), with no recurrence at the 12-month follow-up. It was at this appointment that the clinical features of FFA (Fig 3)—namely, the loss of bilateral eyebrow, frontal band-like recession of hairline, and complete loss of facial vellus hair—were noted by the treating dermatologist, who performed resurfacing and scar revision. Biopsies of the preauricular area showed the complete absence of hair follicles and adnexal structure, consistent with end-stage scarring alopecia (Fig 4).

## Discussion

FFA is a challenging entity, with a marked increase in incidence in the past years.^5^^,^^6^ Epidemiologically, postmenopausal women constitute the majority of patients with FFA. FFA is often asymptomatic but may present with pruritus and, less frequently, trichodynia. The clinical features of FFA consist of band-like scarring alopecia of the frontal hairline, the loss of eyebrows, perifollicular hyperkeratosis, and erythema as well as the loss of facial vellus hair.^5^, ^6^, ^7^ One of the most frequent findings in FFA is eyebrow loss, which has been reported in up to 95% of the cases.^8^ The histology of FFA is identical to that of lichen planopilarus, with a perifollicular lymphohistiocytic inflammatory reaction with progressive follicular fibrosis and the irreversible destruction of stem cells in the bulge area.^2^

The pathogenesis of FFA remains complex and not fully understood.^8^ Currently, the immune-mediated inflammatory mechanism provides the most convincing level of evidence.^5^^,^^8^ The immune-mediated mechanisms involve T cell–mediated attacks and, subsequently, the loss of follicular stem cells. This leads to permanent fibrosis through the inability of hair to regenerate and the epithelial-mesenchymal transition of the follicular stem cell, leading to a fibrotic phenotype. The immune-mediated reaction affects the hair of growth cycle, leading to the loss of vellus, intermediate, and terminal hair. Genetic, hormonal, and environmental factors have also been implicated in the pathogenesis of FFA.^8^

Ablative laser therapy achieves its effect through the ablation of the full-thickness dermis and the subsequent re-epithelialization.^3^ Ablative lasers in skin rejuvenation therapy are well established treatment modalities.^3^^,^^4^ Postprocedural erythema and edema are the most common complications from ablative laser therapy, followed by pain and peeling.^3^ Hypertrophic scarring can occur as a rare complication, with limited cases reported in the literature.^4^ The likelihood that this patient experienced iatrogenic scarring secondary to the laser is low, as the settings were conservative and the patient did not experience postprocedural complications that would increase the risk of scarring, such as infection. Additionally, the patient was compliant with postprocedure care and follow-ups.

The pathogenesis of FFA and the biology of re-epithelialization in wound healing offers a potential explanation for the extensive facial scarring after ablative laser therapy. The re-epithelialization process requires new cells to replace the lost keratinocytes from the initial injury; lineage tracing studies on both human and animal skins have demonstrated the role of hair follicle stem cells in optimal wound healing.^9^^,^^10^ Bulge follicular stem cells are activated upon full-thickness wounding and produce progeny in the interfollicular epidermis for re-epithelialization.^3^ FFA results in the loss or malfunction of the follicular stem cell through a progressive immune reaction and fibrosis. The loss of facial vellus hair observed in this patient, along with the classical features of FFA, suggests the involvement of the facial follicles in the disease process. As the epidermis is ablated by an erbium laser, the optimal re-epithelialization process, which involves the migration and proliferation of follicular stem cells, is impaired due to the lack of pilosebaceous units as a result of the progressive immunological attack and fibrosis of the follicles. The lack of sufficient follicular stem cells leads to extensive scarring. The use of a nonablative laser involves a nonwounding process that creates zones of thermal injury without the obliteration of epidermal or dermal structures. This creates an inflammatory response, leading to increases in matrix metalloproteinase, collagen I, collagen III, and procollagen I, which promote tissue remodeling.^11^ This may explain the excellent response that this patient had with scar revision using a nonablative CO_2_ laser, as the healing process did not require follicular stem cells.

## Summary

FFA is a condition of scarring hair loss, with an increasing incidence being reported in the current literature. It is often a clinical diagnosis and has several distinct clinical features to guide the clinician toward a diagnosis. Laser procedures are increasingly common, with evidence-based efficacy and safety. In this article, we have presented the first known case, to our knowledge, of extensive facial scarring in a patient with undiagnosed FFA after an ablative erbium laser resurfacing procedure. We hope to illustrate the importance of recognizing FFA, or scarring alopecia in general, in patients presenting for laser therapy and avoiding ablative procedures to avoid potentially serious complications that may lead to potentially serious scarring.

## Conflicts of interest

None disclosed.
